# Classification and Morphological Analysis of Vector Mosquitoes using Deep Convolutional Neural Networks

**DOI:** 10.1038/s41598-020-57875-1

**Published:** 2020-01-23

**Authors:** Junyoung Park, Dong In Kim, Byoungjo Choi, Woochul Kang, Hyung Wook Kwon

**Affiliations:** 1 Department of Embedded Systems Engineering, Incheon National University, Incheon, 22012 South Korea; 2 Division of Life Sciences, Incheon National University, Incheon, 22012 South Korea; 3 Convergence Research Center for Insect Vectors, Incheon National University, Incheon, 22012 South Korea

**Keywords:** Entomology, Computer science, Software

## Abstract

Image-based automatic classification of vector mosquitoes has been investigated for decades for its practical applications such as early detection of potential mosquitoes-borne diseases. However, the classification accuracy of previous approaches has never been close to human experts’ and often images of mosquitoes with certain postures and body parts, such as flatbed wings, are required to achieve good classification performance. Deep convolutional neural networks (DCNNs) are state-of-the-art approach to extracting visual features and classifying objects, and, hence, there exists great interest in applying DCNNs for the classification of vector mosquitoes from easy-to-acquire images. In this study, we investigated the capability of state-of-the-art deep learning models in classifying mosquito species having high inter-species similarity and intra-species variations. Since no off-the-shelf dataset was available capturing the variability of typical field-captured mosquitoes, we constructed a dataset with about 3,600 images of 8 mosquito species with various postures and deformation conditions. To further address data scarcity problems, we investigated the feasibility of transferring general features learned from generic dataset to the mosquito classification. Our result demonstrated that more than 97% classification accuracy can be achieved by fine-tuning general features if proper data augmentation techniques are applied together. Further, we analyzed how this high classification accuracy can be achieved by visualizing discriminative regions used by deep learning models. Our results showed that deep learning models exploit morphological features similar to those used by human experts.

## Introduction

Mosquitoes cause global infectious disease burden, as vectors of numerous fatal diseases like malaria and dengue thrive by climate change and insecticide resistance. For example, in 2017, an estimated 219 million cases of malaria occurred and an estimated 435,000 deaths from malaria globally^1^. For this reason, there have been significant efforts to develop a surveillance system for early detection and diagnosis of potential outbreaks of mosquito-borne diseases. Although, mosquito monitoring programs have been intensively developed worldwide, current mosquito monitoring procedures have many limitations. In particular, it takes at least a few days to detect potential pathogens of mosquito-borne diseases. Furthermore, one of the major bottlenecks in mosquito monitoring is that even classification by human experts of collected mosquitoes is a laborious and time-consuming procedure. Traditionally, trained researchers or technicians classify the species of mosquitoes by visual examination of morphological keys that provide step-by-step instructions on taxonomic characteristics of a given mosquito species^2^. However, the number of taxonomists and classification experts has drastically decreased so far^3^. Therefore, alternative automatic identification methods with expert-level classification accuracy are highly required in this field.

Automatic classification of species is important not just for mosquitoes, but also for insects in general since it contributes to various purposes such as environment monitoring, pest diagnostics, border security, forensics, and vector epidemiology, etc. Previous approaches have used various properties of mosquitoes and insects for automatic species classification, including 3D images^4^, sound^5^, genomic data^6^, etc. For example, Banerjee *et al*.^6^ proposed to exploit artificial neural system for classification and identification of *Anopheles* mosquito species based on the information content of ribosomal DNA sequences. Fanioudakis *et al*.^7^ acquired light amplitude variation of flight recording cases from 6 mosquito species, and trained a deep learning model to achieve 96% classification accuracy. However, the data acquisition steps of these previous work are laborious and time-consuming and, hence, cannot be performed in real-time. In contrast, 2D images are relatively easy to acquire, and, in this context, the application of computer vision using 2D images has been studied as a promising approach to automated entomology.

Image-based systems perform three sequential steps for species classification: 1) image acquisition, 2) feature extraction and c) classification. However, there are several challenges in these steps. First of all, mosquitoes and insects usually manifest wide variations in pose and deformation aside from classical image variations such as scale, rotation, lighting and cluttered background. For example, many image-based automated classification methods rely on geometry of wing venation^8,9^. However, capturing images of wings in a constrained pose is also time-consuming and laborious process as the traditional recognition procedure. Furthermore, wings and other appendages such as legs can be easily damaged during the process. Due to the difficulty of image acquisition, the availability of datasets is tremendously limited, particularly in same families such as Cullicidae (mosquitoes). The datasets used in previous work usually have less than dozens of images per species^8,9^.

Another difficulty lies in obtaining proper features from images. Early work on insect and mosquito classification have used global representation based on color, texture, color histogram, geometrical shapes (e.g., area, perimeter, holes number, eccentricity and roundness)^10–12^, wavelet coding^13^, or other relatively simple features^14,15^. Some examples of systems using such techniques include the automated bee identification system (ABIS)^15^, and digital automated identification system (DAISY)^16^. These approaches usually require considerable domain expertise to design feature extractors. However, these precisely extracted features are easily affected by factors such as the pose of specimens, scale, lighting, etc. To remedy this problem, recently, local feature-based approaches have gained popularity. In particular, ‘bag-of-visual-words’ framework is a powerful local feature-based method. Local features extracted using local operators, such as Histogram of Oriented Gradients (HOG)^17^ and Scale Invariant Feature Transforms (SIFT)^18^, are encoded into visual words to represent each image as a frequency of histogram of features in the image. Once intermediate image representation using visual words are constructed, a classifier is trained to classify objects. While histogram of visual words has been applied successfully to many applications^4,19,20^, they rely on careful choice of feature size.

More recently, since a deep learning model, called AlexNet^21^, won 2012 ImageNet Large Scale Visual Recognition Challenge (ILSVRC) competition^22^ by a wide margin, deep learning models such as Deep Convolutional Neural Networks (DCNNs) have demonstrated state-of-the-art classification performance in many recognition tasks including document recognition^23^, age and gender classification^24^, defect pattern classification in semiconductor manufacturing, galaxy morphology prediction^25^, and vehicle type classification^24^, to name a few. A DCNN architecture consists of multiple layers of non-linear operations that are highly effective to represent hierarchical features, and address many drawbacks of previous image-based recognition techniques. Combined with proper training methods such as regularization, these hierarchical features are automatically learned during training process, alleviating the needs for designing feature extractors manually. Further, these learned feature extractors have demonstrated remarkable robustness to variation of input images. Inspired by the great success of DCNNs, there have been great efforts to apply DCNNs to automatic entomology. For instance, Liu *et al*. trained a variant of AlexNet to classify paddy field pests and achieved 0.951 classification accuracy^26^. Liu *et al*. collected over 5,000 images of 12 species from the Internet. Zhu *et al*.^27^ classified 22 species of lepidopteran species by combing a DCNN and a supported vector machine algorithm. These previous work classify insect species belonging to different families, thereby having distinct differences.

In this study, we investigate DCNNs for their ability to overcome the challenges in mosquito species classification tasks. We are particularly interested in exploring DCNN’s ability to classify mosquitoes having high inter-species similarity and intra-species variations. For our test, we use 8 species of the three major genera of disease vectors: *Anopheles*, *Aedes*, and *Culex*. We are also interested in testing if visual features captured by DCNNs match the morphological keys used by human experts. To address these questions and challenges, we make the following contributions:

1. *Dataset for fast image acquisition of specimens:* Learning deep hierarchical representations in DCNNs requires a large amount of images. Unfortunately, not many datasets are available for mosquito species. Most available datasets have flatbed wing images^8,9^, which are hard to acquire by non-experts. For the ease of image acquisition, we have built a dataset with about 3,600 images of 8 mosquito species that have various poses and deformation conditions (e.g., missing body parts).
2. *Transfer and fine-tuning of features learned from generic dataset:* Even though our dataset has about 3,600 images for eight mosquito species, it is not enough to train state-of-the-art DCNN architectures, such as ResNet^28^ and VGGNet^29^. To address this limitation, we apply the transfer learning paradigm in which representation gained on larger generic dataset is transferred to recognize mosquitoes. Three state-of-the-art pretrained DCNN models are fine-tuned to take the benefit of hierarchical representation learned from the generic ImageNet^22^ dataset.
3. *Visualization of morphological features:* The learning process of DCNNs is end-to-end, from raw images to final mosquitoes species. Therefore, unlike traditional handcrafted feature extractors, it is very hard to get insight into internal operations. Without clear understanding of what properties of mosquitoes are used for the classification of species, the development of better classification method is impossible. To address this problem, we introduce a recently developed visualization methods^30,31^ that localize discriminative regions of mosquitoes at each convolution step. With this visualization method, we compare the discriminative regions detected by DCNN models with the morphological keys used by human experts.

Our experiment results show that more than 97% classification accuracy can be achieved by fine-tuning pretrained DCNNs if proper data augmentation techniques are applied for further retraining. Our results also demonstrate that the discriminative regions identified by DCNNs are well-matched to some morphological keys used by human experts. We anticipate that our experimental results will inspire further research on image-based automatic classification of mosquito species for early detection of potential vector-borne diseases.

## Vector Mosquitoes Dataset

The first goal of this study is to collect a large labeled image dataset of female vector mosquitoes to facilitate the automatic classification of their species. To this end, we selected five representative mosquito species that are known as major vectors of diseases such as Japanese encephalitis, dengue, and Zika. Table 1 shows these five target species and their vector diseases. We also used three additional mosquito species that are often confused with the target vector mosquito species. We grouped these three mosquito species into a single *less-potential* class since they are considered as less potential vectors transmitting infectious diseases. About 120 samples per species were captured in various locations in South Korea. Two species (*Aedes albopictus* and *Culex pipiens*) were bred in a mosquito insectary of Incheon National University, and the others (*Aedes vexans*, *Aedes dorsalis*, *Anopheles* Spp. *Aedes korekus*, *Culex inatomii*, *Culex pipiens*) were captured in the field.

**Table 1:** Summary of the mosquito dataset.

Species	Vector Disease	Captured Location	# of images
*Ae*. *albopictus*^†^	Zika, Dengue	Jong-no Seoul	600
*Ae*. *vexans*	Zika, Westnile Virus	Tanhyeon Paju	591
*Anopheles* spp.	Malaria	Tanhyeon Paju	593
*Cx*. *pipiens*^†^	Westnile virus	Jong-no Seoul	600
*Cx*. *tritaeniorhynchus*	Japanese Encephalitis	Tanhyeon Paju	594
*Ae*. *dorsalis*	—	Tanhyeon Paju	200
*Ae*. *koreikus*	—	Tanhyeon Paju	200
*Cx*. *inatomii*	—	Tanhyeon Paju	200

Species marked with ^†^ are captured at the lab. facility. We treat *Ae*. *dorsalis*, *Ae*. *koreikus* and *Cx*. *inatomii* as a single *less-potential* class.

Since we were aiming to facilitate fast classification of mosquitoes by non-experts, the mosquito images of the dataset should reflect variations typically found in field-captured mosquitoes. For example, aside from classical image variations, mosquitoes have highly variable poses. Further, mosquito samples can be easily damaged, discolored, and lose morphological characteristics during the capture in the field and the process of preservation through freezing and drying. Due to the unavailability of dataset satisfying such requirements, we took mosquito images from the samples using a digital microscope (Nahwoo Pixit FHD One). Since the number of mosquito samples is limited, we took about 3–5 images for each specimen by physically varying the postures, angles, and the light intensity. Each original image has the resolution of  $2952\times 1944$ pixels with 24 bit RGB channels. The original images are resized to lower resolutions of $420\times 314$ pixels for the ease of image-acquisition with typical off-the-shelf cameras. Through this manual image acquisition process, we collected about 600 images for each target vector mosquito species, totaling about 3,000 images, in addition to 600 more images for three additional less-potential vector mosquito species.

## Deep Convolutional Neural Networks

### DCNN model architecture

The second goal of this work is to establish the baseline accuracy expected from modern deep learning models when classifying vector mosquitoes with high inter-species similarity and intra-species variations. We were particularly interested in investigating the effectiveness of transferring features learned from a generic dataset into the classification of vector mosquitoes. To this end, we exploited 3 representative, but contrasting, off-the-shelf DCNN models shown in Table 2. VGG-16 represents relatively shallow, but memory-intensive deep learning models that have a large number of parameters^29^. Even though VGG-16 has only 16 layers, its three fully-connected layers occupy more than 90% of its 138 million parameters. In contrast, ResNet-50 represents deep and highly computation-intensive deep learning models^28^. Even though ResNet-50 has deep 50 layers, it requires only 25.6 million parameters. ResNet-50 achieves the state-of-the-art DCNN performance with about 94.75% classification accuracy on ImageNet. Finally, SqueezeNet^32^ is a light-weight DCNN model that allows real-time classification in resource-constrained mobile and embedded devices^33^. Table 2 summaries the characteristics of these three models. Throughout this paper, VGG-16 is used to discuss the model training and visualization process because its architecture is relatively simple and intuitive. However, our discussion with VGG-16 can be extended to other deep learning models without loss of generality.

**Table 2:** The summary of DCNN architectures investigated in this work.

DCNN Model	# of Layers (#Conv. + #FC)	# of Parameters (×10^6^)	Top-5 accuracy on ImageNet-2012	Latency [ms]	Energy/Inference [mWh]
VGG-16	13 + 3	138	90.4	98.63	0.32
ResNet-50	49 + 1	25.6	94.7	40.18	0.14
SqueezeNet	18 + 0	1.23	80.3	13.26	0.03

The specification of the DCNN models are from their respective original papers^28,29,32^ except the inference latency and energy consumption. For consistency, the inference latency and energy consumption are all measured in the Nvidia Jetson TX2 embedded device.

**Figure 1:**
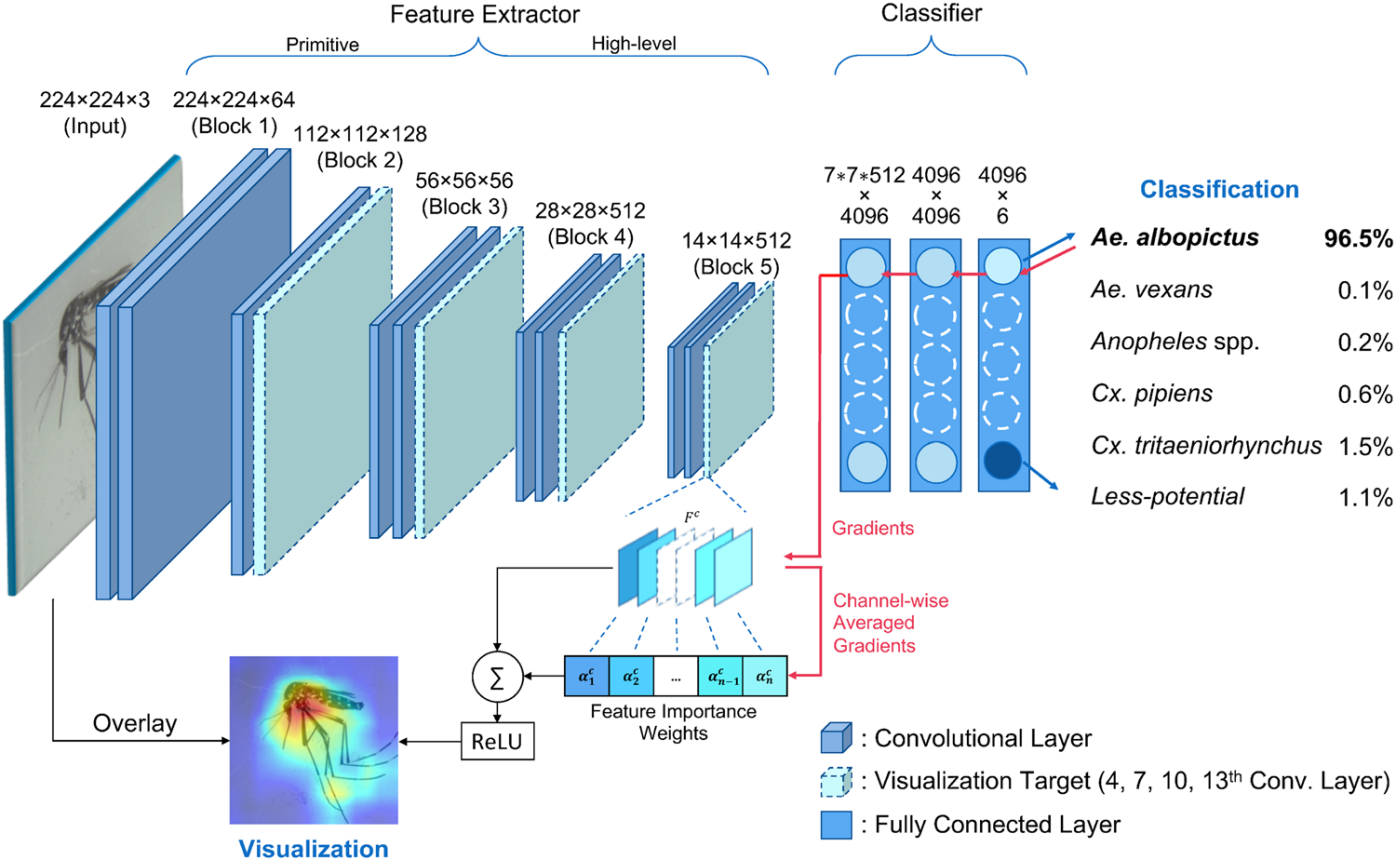
The flow of the classification and visualization in the VGG-16 DCNN model. The class of a given mosquito image is predicted by two steps: (1) extracting hierarchical features and (2) classifying these features. In the feature extractor part, feature maps generated by filters at each convolution layer are shown. These feature maps are used for visualization by weighting them with channel-wise averaged gradients.

Figure 1 shows the steps of transforming an input image into final classification results in VGG-16. These steps are divided broadly into 2 parts: *feature extraction* and *classification*. At the *feature extraction* part, a series of convolution layers apply a set of filters to input images (or features maps) to detect the presence of particular patterns, or *features*. For instance, the first convolution layer extracts features from 224 × 224 × 3 input images using 64 filters of $3\times 3$ spatial dimensions to generate activation maps of $224\times 224\times 64$. The activation maps, often called *feature maps*, are a collection of feature activation detected by neurons at each convolution layer and have the dimension of $height\times width\times channels$. These feature maps are processed successively by the next convolution layer as an input to detect the higher level features. By applying several convolution layers in succession, the spatial dimensions of feature maps are reduced gradually from $224\times 224$ to $14\times 14$, so that the neurons in deeper convolution layers detect patterns in broader spatial areas of input images. For instance, while the filters in the shallow layers are trained to detect primitive features such as edges and colors within their receptive fields, the filters in the deep layers learn more abstract and high level features such as overall shapes and patterns of wings, legs, and bodies using low level features. In later sections, we visualize these feature maps by highlighting them according to their importance for correct classification. The feature maps at the final convolution layer have $14\times 14\times 512$ dimensions, and are flattened into a one-dimensional vector for the classification by the three fully connected (FC) layers. Since the original DCNN models were designed to classify 1,000 classes, the final fully connected layer had $4096\times 1000$ dimensions. We replaced this layer to have $4096\times 6$ dimensions to classify 6 mosquito classes. Each target vector mosquito species was assigned to a separate class, and three additional less potential species were grouped into a single *less-potential* class. The outputs of the final fully connected layers ${y}^{c}$ are the scores for the class $c\in [\mathrm{1..6}]$. These scores ${y}^{c}$ are processed by *softmax* operations to show the classification probability ${p}^{c}$ of each mosquito species class *c*.

### Training the deep convolutional neural networks

Training is an iterative process of learning features by minimizing the error between the model predictions and the labeled training data. Instead of training the models from scratch, we *fine-tuned* the models trained on generic dataset since our mosquito dataset was not enough for training complex models such as VGG-16^34^. Hence, we first loaded the model parameters pre-trained on ImageNet. The final fully connected layer was initialized by the uniform random distribution in the range of $[\,-\,15e-4,15e-4]$. We used ADAM^35^ as an optimizer with parameters ${\beta }_{1}$ and ${\beta }_{2}$ respectively set to 0.9 and 0.999. The general cross-entropy loss function was used for training. The dataset was partitioned into 80–20% splits of training and test datasets. The training was performed only on the 80% training dataset and the remaining 20% dataset was reserved for testing. We further applied 5-fold cross validation approach. Hence, the training data was partitioned randomly into 5 partitioned sub-groups, and one of them was held as a validation dataset while the others were used for training. The validation dataset was used to monitor the progress of training.

Despite our effort of capturing variability of mosquito species, most state-of-the-art DCNN architectures with many layers typically require much more training data for stable performance. To overcome the lack of labeled data, we also applied a series of data augmentation techniques to both the training and the test images. After normalizing the images to have [0, 1] range of pixel values, all images were randomly rotated in the range of [$0^\circ ,360^\circ$] degrees. Then, they were scaled both vertically and horizontally in the range of ±15% with the same aspect ratio. The brightness of the images was randomly adjusted in the range of ±10%. Moreover, in consideration of various lighting condition in common lab environments, we also applied random shifts of hue in the range of ±10%, contrast and saturation shift in the range of ±20%. Finally, we cropped a $224\times 224$ patch from the center of the image to meet the input sizes of deep learning models.

**Figure 2:**
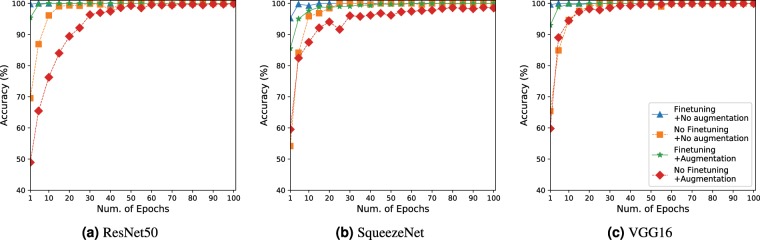
Validation accuracy of the models during the training. In all models, optimal validation accuracy was reached early when both the data augmentation and fine-tuning were applied together.

For model training and evaluation, we used PyTorch deep learning framework on an Nvidia 2080Ti GPU platform. As shown in Table 3, different initial learning rates were set for different models, ranging from $3e-6$ to $7.5e-3$. The training was performed for 100 epochs and the learning rates are reduced by 0.25 every 15 epochs. Figure 2 illustrates the training process for the chosen models. To show the effectiveness of fine-tuning and data augmentation, we also trained the same models while either or both fine-tuning and data augmentation were not applied. As shown in Fig. 2, the validation accuracy of most models with these settings reached to plateau within 30 epochs and achieved optimal validation accuracy within 100 epochs.

**Table 3:** The average test classification accuracy.

DCNN model	Augmentation	Fine-tuning	LR	Accuracy (%)
VGG-16			5e-6	38.96
		✓	5e-6	56.76
	✓		5e-6	91.15
	✓	✓	5e-6	**97**.**19**
ResNet-50			5e-3	57.47
		✓	1e-3	57.74
	✓		5e-3	93.45
	✓	✓	7.5e-3	**96**.**86**
SqueezeNet			3e-5	47.42
		✓	3e-5	62.81
	✓		3e-5	78.58
	✓	✓	3e-5	**90**.**71**

Each model is trained with four settings of dynamic data-augmentation and fine-tuning. When a model is not fine-tuned, it is trained from scratch with random initialization.

### Classification performance

Table 3 shows the average classification accuracies on the test dataset. Even though all approaches reached almost 100% validation accuracy in about 100 epochs in Fig. 2, the test accuracies were much lower than the validation accuracies for most models and settings. For instance, the test accuracy of VGG-16 was only 38.96% when neither data augmentation nor fine-tuning was applied. The highest performance 97.19% was achieved by VGG-16 when both the data argumentation and fine-tuning were applied. This gap between the validation accuracy and the test accuracy implies that overfitting was occurred during the training. This was an expected result because the data in the training dataset was used both for training and validation with the *k*-fold cross validation. In contrast, test dataset was never used for the training process, and, hence, the test classification accuracy revealed true baseline accuracy expected from state-of-the-art DCNN models.

It should be noted that all models achieved significantly higher test accuracy when the data augmentation was applied. For example, VGG-16, SqueezeNet, and ResNet-50, respectively, achieved about up to 52.2%, 31.1%, 35.9% higher test accuracy when data augmentation was applied. Fine-tuning had different effects for different models. For VGG-16, applying fine-tuning increased the test accuracy by up to 17.8%. In contrast, ResNet-50 had only up to 3.4% increase of test accuracy when fine-tuning was applied together with the data argumentation. We believe that proper initialization of a pre-trained model is very beneficial for proper training of VGG-16, since VGG-16 has a larger amount of parameters than other models.

In the remaining sections, we choose to use the VGG-16 model trained with data augmentation and fine-tuning since it achieved the highest test accuracy.

### Effectiveness of transferring features

*Transfer learning* is a common technique in deep learning to overcome the data scarcity problem since training from scratch very deep networks is not viable without a huge amount of data. With transfer learning, off-the-shelf features extracted from pre-trained networks are reused for new target tasks. The basic idea behind transfer learning is that shallow features are generic while deep ones are more specific to the source task^36,37^. To investigate the effectiveness of off-the-shelf features in the classification of mosquito species, we applied several different fine-tuning strategies as shown in Table 4.

**Table 4:** The test accuracies with different partial fine-tuning strategies for VGG-16.

Model	Fine-tuning targets	Accuracy(%)
VGG-16 - **M1**	All FC layers	76.05
VGG-16 - **M2**	5th Conv. block + All FC layers	87.26
VGG-16 - **M3**	4–5th Conv. blocks + All FC layers	88.51
VGG-16 - **M4**	2–5th Conv. blocks + All FC layers	93.11
VGG-16 - **ALL**	All Conv. blocks and FC layers	97.19

In all settings, models are initialized with pre-trained weights from the ImageNet dataset. During the training with the ADAM optimizer, the learning rates are set to 5e-6 and reduced by 0.25 every 15 epochs.

We started from a VGG-16 model pre-trained with the ImageNet dataset, and replaced the final fully connected layer to match the number of target mosquito classes. During the re-training for 100 epochs, we froze several layers of the model and fine-tuned only the remaining layers with our mosquito training dataset. Table 4 shows 5 different fine-tuning settings. For instance, in VGG-16-M1, only the classifier part with three fully connected layers was retrained while the feature extractor part with convolution layers was frozen. This implies that all features learned from generic ImageNet dataset were reused without modification in VGG-16-M1. In contrast, in VGG-16-ALL, all layers were fine-tuned via re-training, and this was the default setting used throughout this work.

**Figure 3:**
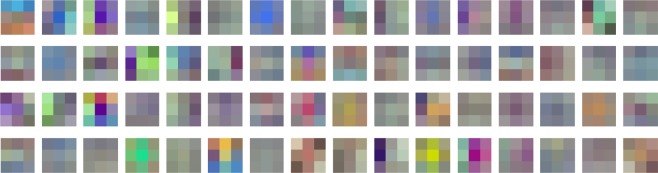
The visualization of 64 filters of the first convolution layer in the *VGG-16* model. Filters with $3\times 3\times 3$ dimensions are projected into pixel space for the visualization.

Table 4 shows the results. Our first observation revealed that transferring deep features without fine-tuning is not effective. For example, the test accuracy of VGG-16-M1 was only 76.05%, which is much lower than the test accuracy of 91.15% of models trained from scratch as shown in Table 3. In contrast, shallow features learned from ImageNet dataset were much more useful for the classification of mosquitoes. As shown in Table 4, as we increased the fine-tuned layers gradually, the classification accuracy also increased rapidly. For instance, when only the features from the first convolution block (or 2nd and 3rd convolution layers) were reused in VGG-16-M4, the test accuracy of 93.11% was achieved. This result demonstrates that shallow features are more generic since they capture primitive patterns such as edges, arcs, and colors. Figure 3 shows the visualized filters of the first convolution layer of VGG-16-ALL. These patterns in the filters seem to be very generic and have no peculiarity to mosquitoes, at least to human eyes. After careful numerical comparison with the original filters, we found only slight differences in the colors of the filters. We believe that this is because the images in the ImageNet dataset are more likely to be colorful than our mosquito images. Finally, it should be noted that the highest classification accuracy was achieved when all layers were fine-tuned as shown in Table 4. This result demonstrates that features learned from ImageNet dataset are generally useful to overcome the scarcity of data, but overall fine-tuning is required to capture mosquito-specific features.

## Identification of Morphological Keys Used by DCNNs

In previous sections, we demonstrated that state-of-the-art DCNNs were able to achieve high classification accuracy for mosquito species. However, it is still unclear how this high accuracy can be achieved since DCNNs learn features through end-to-end learning, excluding human expertise for feature engineering. It also raises a question of whether DCNNs use similar morphological keys used by human experts to classify mosquito species. In this section, we apply recent visualization techniques to identify the mosquito regions used by DCNNs and compare them with the morphological keys used by human experts.

### Visualization of feature activation

As shown in Fig. 3, the first convolution layer learns primitive features such as edges, arcs and colors. In contrast, deep intermediate layers are supposed to learn more complex and abstract features. However, the visualization technique used for the first convolution layer is not applicable for deep intermediate layers since these layers have many channels and, hence, the visualized filters do not give intuitive interpretation for human understanding. Instead, recent works in computer vision have demonstrated that visualizing feature activations gives more intuitive results for intermediate layers of DCNNs^30,31,37,38^. By visualizing feature activations, we can identify which regions of the input images contribute to the classification results. In this work, the feature visualization techniques can be used to identify body parts of mosquitoes used by DCNNs to classify similar mosquito species.

The visualization of feature activations of a convolution layer was done by projecting weighted feature maps onto the original input image. Since each element of a feature map reflects the activity of a neuron on particular lower level features, the elements of a feature map were weighted according to their contribution to the class score ${y}^{c}$, as shown in Eq. (1). When a convolution layer had $n$ feature maps of $u\times v$ spatial dimensions, each element of *k*-th feature map ${A}_{k}$ was weighted according to the importance factor ${\alpha }_{k}^{c}$. The importance factor ${\alpha }_{k}^{c}$ of *k*-th feature map can be set to reflect the contribution of the feature map to the classification for class *c*. Though ${\alpha }_{k}^{c}$ can be estimated in several different ways, in this work, we used the gradient of the class score ${y}^{c}$ with respect to feature map ${A}^{k}$, as shown in Eq. (2) ^31^. These gradients were global average-pooled to obtain the channel importance of *k*-th feature map on the prediction of *c*-th class.

1 $${L}^{c}=ReLU\left(\mathop{\sum }\limits_{k}^{n}\,{\alpha }_{k}^{c}{A}^{k}\right)$$

where,

2 $${\alpha }_{k}^{c}=\frac{1}{u\times v}\,\mathop{\sum }\limits_{i}^{u}\,\mathop{\sum }\limits_{j}^{v}\,\frac{\partial {y}^{c}}{\partial {A}_{i,j}^{k}}$$

In Eq. (1), *ReLU* non-linearity operations were applied to the weighted feature maps to activate only features that have positive influence on the prediction. Since ${L}_{i,j}^{c}$ indicates the importance of the activation of the neuron at spatial grid $(i,j)$, $L$ can be visualized as a heatmap to better show the discriminative regions in the input images. Before the projection of $L$ onto an input image, it needs to be resized to match the sizes of input images. As shown in Fig. 1, the feature maps $A$ of deeper convolution layers have smaller spatial dimensions, and, hence, they capture features in the broader area of input images. For instance, the receptive fields of a neuron at the 4-th, 7-th, 10-th, and 13-th convolution layers are supposed to detect the features, respectively, in 6 × 6, $12\times 12$, $24\times 24$, and $48\times 48$ sub-areas of the input images. Therefore, when ${L}^{c}$ is visualized as a heatmap by overlaying on input images, the deeper convolution layers display increasingly broader discriminative regions.

**Figure 4:**
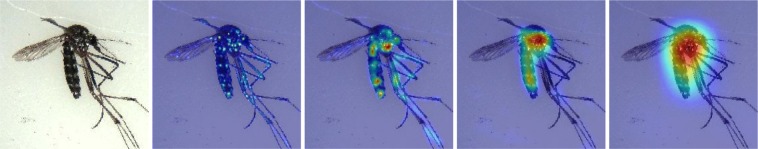
The visualization of discriminative regions in the 4-, 7-, 10- and 13-th convolution layers of VGG-16 when an *Aedes albopictus* input image is given.

Figure 4 shows as heatmaps of the visualized feature maps of a few chosen convolution layers when an image of *Aedes albopictus* is given as input. In the heatmaps, important regions are displayed in red colors. Since morphological characteristics of mosquitoes have different scales, we need to visualize several layers, not just the final convolution layer. For instance, the heatmap of the 13-th convolution layer, which is the final convolution layer, localizes coarse-grained discriminative regions. The heatmap of the 13-th convolution layer in Fig. 4 highlights the lateral thorax of the sample as the most important region for classifying the sample into the *Aedes albopictus* class. In contrast, heatmaps of the shallow layers localize more fine-grained features. For instance, the heatmap of the 7-th convolution layer shows that the striped pattern in the abdominal tergite and legs (femur and tarsus) are important for the classification.

### Comparison of morphological keys with DCNN’s discriminative regions

**Figure 5:**
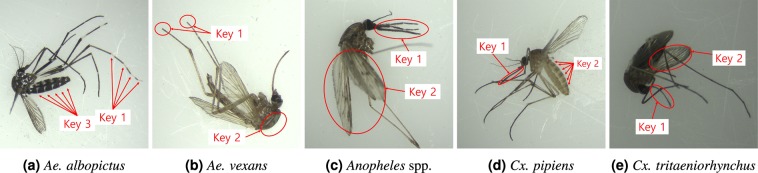
Visual keys used by human experts are marked with red circles and arrows. Each key is numbered according to the list of keys in Table 5. Some evident or invisible keys are not shown.

The classification of mosquito species using morphological keys has been studied extensively not just for academic purposes but also for practical purposes such as epidemiological activities of public health workers^2^. These pictorial keys provide step-by-step guides to classify mosquito species having high inter-species similarity. In Table 5, a few notable keys used by human experts are summarized^2^. These keys are mostly about the colors and patterns of body (scutum and abdomen), legs (typically tarsi), proboscis, and the venation in wings. In Fig. 5, these keys are depicted for the target vector mosquito species. Some keys, such as the abdominal bands of *Aedes vexans*, are not marked on the images due to the poor condition of the samples.

**Table 5:** Morphological keys used by human experts for classifying vector mosquito species.

Species	Morphological keys used by human experts	Highlighted in heatmaps?
*Aedes albopictus*	1. Tarsi with pale bands	Sometimes
	2. Body relatively darker than the other species	Mostly
	3. Abodominal terga II-VII with large laterobasal patches	Mostly
*Aedes vexans*	1. Last segment of mid and hind tarsi dark apically	Sometimes
	2. Scutum yellowish brown without patches or stripes	Mostly
	3. Middle abdominal bands B-shaped	Rarely
*Anopheles* spp.	1. Palpus as long as proboscis	Sometimes
	2. Wing spotted	Mostly
*Culex pipiens*	1. Proboscis without a pale band	Rarely
	2. Abdomen with basal bands	Mostly
	3. Body yellowish brown	Mostly
*Culex tritaeniorhynchus*	1. Proboscis with a pale band in the middle	Rarely
	2. Costa and other veins without pale band	Sometimes
	3. Body relatively small and reddish brown	Mostly

Most morphological keys related to the body area are highlighted actively in the heatmaps.

**Figure 6:**
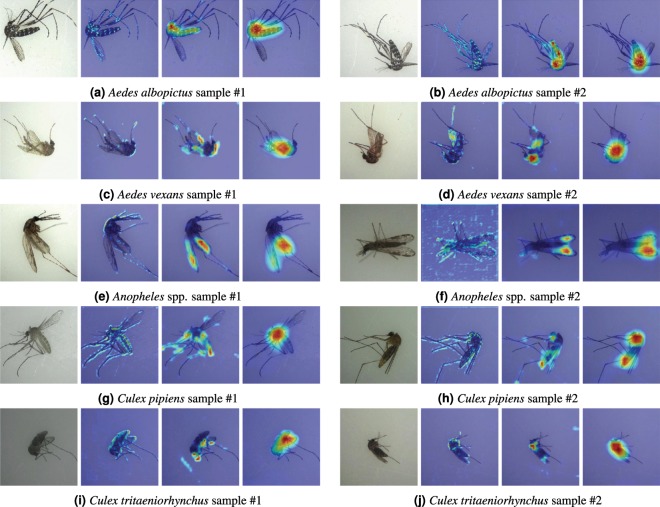
The visualization of 5 vector mosquito species. Discriminative regions captured by DCNNs are shown in heatmaps for shallow (4-th), middle (8 or 9-th), and deep (12-th) convolution layers.

Figure 6 shows two samples for each target species with their heatmaps of feature activation. We compared the morphological keys used by human experts with these discriminative regions captured by DCNNs. For better comparison, the same sample images in Fig. 5 were used as the *sample #1* for each species. In Table 5, we show how often the keys used by human experts are highlighted in the heatmaps.

First, *Aedes albopictus* is an epidemically important vector for the transmission of many viral pathogens, such as yellow fever virus and dengue fever, and is relatively easy for human observers to identify because its body is relatively darker than other species^39^. *Aedes albopictus* is called a tiger mosquito for its striped appearance; it has white bands on its legs and body^40^. Our samples of *Aedes albopictus* were captured from laboratory-reared colonies, and, hence, they were in a relatively good condition without much damage to the bodies, showing all typical morphological keys listed in Table 5. With the visualization of specimens, we found that some morphological keys used by human experts were also very strong discriminators for DCNNs. As shown in Fig. 6(a,b), the dark bodies (**key 2**) and the patches in abdominal terga (**key 3**) are strongly highlighted in both samples. The pale bands on the legs (**key 1**) are slightly highlighted in the heatmaps of shallow convolution layers, but they are not as strong as the characteristics of the bodies.

*Aedes vexans* could serve as a potential vector for Zika virus in northern latitudes^41^, and it can be recognized by yellowish brown bandless scutum and B-shaped markings on each abdominal tergite when viewed sideways. We found that abdominal tergite is not visible in many specimens because the samples were captured in the field and their abdominal parts were often dried and contracted. This situation usually occurs if the specimens are mishandled after caught in the wild. Despite the poor condition of these specimens, as shown in Fig. 6(c,d), the yellowish brown color of the bodies and scutum (**key 2**) serve as very strong discriminators of *Aedes vexans*. As expected, the heatmaps show that the abdominal terga (**key 3**) are not actively used by DCNNs to classify *Aedes vexans*. Due to the low resolution of the images, dark apical tarsi (**key 1**) are not easy to recognize even for human experts. However, surprisingly, the heatmap of the shallow layer in Fig. 6(c) shows that they are active discriminators used by DCNNs to classify *Aedes vexans*.

The genus *Anopheles* is the only mosquito taxon known to transmit human malarial protozoa^42^. Since species in *Anopheles* genus are extremely similar morphologically and can only be reliably separated by microscopic examination of the chromosomes or DNA sequencing^43,44^, we grouped the species of *Anopheles* spp. as a single class without further taxonomic separation. Usually, human experts examine wing venation and long palpus to identify *Anopheles* spp. The heatmaps in Fig. 6(e,f), demonstrate that DCNNs also used wing venation (**key 2**) as a strong discriminator to separate *Anopheles* spp. from other species. In contrast, long palpus (**key 1**) was not used by DCNNs as an active discriminator.

*Culex pipiens* is a vector for diseases, including Japanese encephalitis, West Nile virus, Emilia-Romagna, and Usutu virus^45^. *Culex pipiens* is identified by its light golden brown body scales and abdomen distinctly marked with pale broad rounded bands. Since our specimens of *Culex pipiens* were captured from laboratory-reared colonies, they had good condition and showed all these morphological keys. As shown in the heatmaps in Fig. 6(g,h), DCNNs also classified *Culex pipiens* using these characteristics of the body (**keys 2 and 3**). Even though most specimens of *Culex pipiens* had good wing conditions, their wings were hardly used by DCNNs, unlike *Anopheles* spp.

*Culex tritaeniorhynchus* is the main vector of the disease Japanese encephalitis and it has relatively small reddish brown body. *Culex tritaeniorhynchus* can also be identified by dark scaled proboscis with narrow median pale ring. Wing veins of *Culex tritaeniorhynchus* are entirely dark scaled. Since our specimens of *Culex tritaeniorhynchus* were collected from the field, most of them had damages to the legs and proboscis. As a result, the bands in proboscis (**key 1**) were hardly used by DCNNs as shown in Fig. 6(i,j). However, despite these damages, as shown in Table 6, DCNNs showed remarkably high classification accuracy of 99.8%. The heatmaps show that the characteristics of the body (**key 3**) and the wing veins (**key 2**) made a significant contribution to the classification of *Culex tritaeniorhynchus* species.

**Table 6:** The confusion matrix (%) achieved by the VGG-16 model on the test dataset.

	*Ae*. *albopictus*	*Ae*. *vexans*	*Anopheles* spp.	*Cx*. *pipiens*	*Cx*. *tritaeniorhynchus*	*Less-potential*
*Ae*. *albopictus*	**99**.**8**	0.0	0.0	0.0	0.0	0.2
*Ae*. *vexans*	0.0	**96**.**6**	0.8	0.4	2.2	0.0
*Anopheles* spp.	0.0	0.0	**99**.**4**	0.6	0.0	0.0
*Cx*. *pipiens*	0.0	0.0	0.0	**99**.**15**	0.85	0.0
*Cx*. *tritaeniorhynchus*	0.0	0.2	0.0	0.0	**99**.**8**	0.0
*Less-potential*	0.61	6.26	0.0	1.01	0.4	**92**.**53**

Finally, it should be noted that the visualization of mosquito specimens indicates that DCNNs mostly capture the characteristics (e.g., color, size, and shape) in the body area. For instance, the wing patterns were used only for *Anopheles* spp. and *Culex tritaeniorhynchus* while the body patterns played as dominant discriminators in most species. We also found that the features related to the legs, proboscis and palpi were rarely used as dominant discriminators. We believe that this is because of the damages of many field-collected specimens and the low resolution of input images.

#### Analysis of misclassified cases

Even though our DCNNs achieved remarkably high classification performance, some misclassified cases were still found. Table 6 shows the confusion matrix resulting from the VGG-16 model. All vector mosquito species were classified with the test accuracy greater than 96.6%. In contrast, the *less-potential* vector class showed the lowest accuracy of 92.53%. Since the *less-potential* class had 3 mosquito species (*Ae*. *dorsalis*, *Ae*. *koreikus*, and *Cx inatomii*) in a single class, it might have been difficult to capture representative features for the class.

**Figure 7:**
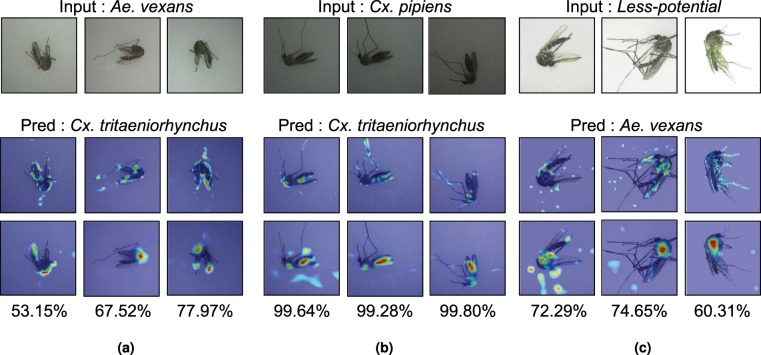
Three major cases of misclassification. Samples are shown with their heatmaps (feature activation at the 7th and 10th convolution layers) and the classification probabilities.

In Table 6, our VGG-16 model confused 2.2% *Aedes vexans* with *Culex triaeniorhynchus*, 0.85% *Culex pipiens* with *Culex triaeniorhynchus*, and 6.26% *less-potential* vector mosquitoes with *Aedex vexans*. Figure 7 shows some samples of such misclassified cases with their heatmaps and prediction probabilities. After careful visual examination of misclassifed cases, we found two major causes of such confusion. The first reason for the confusion was the bad condition of field-captured specimens. For instance, as shown in Fig. 7(a), *Aedes vexans* were often confused with *Culex triaeniorhynchus* when the specimens were badly damaged. Most misclassifed *Aedes vexans* specimens had only a few legs and their bodies were distorted and discolored. With such severe damages, it is challenging even for human experts to classify them correctly. Another reason of frequent confusion was the lighting condition when images were taken. For example, the confusion, shown in Fig. 7(b,c), resulted from the effect of lighting. As noted in previous sections, one of the most important discriminators of *Culex pipiens* was its yellowish brown body color. But, if the images were too dark to distinguish the body color, they were often confused with *Culex triaeniorhynchus*, whose body is dark brown. Too much light also degraded the classification performance. As shown in Fig. 7(c), when the light was too bright, some *less-potential* mosquitoes were often confused with *Aedes vexans*, whose body is yellowish brown.

## Conclusions

In the present study, we demonstrated the effectiveness of deep convolutional neural networks for classifying vector mosquito species having high inter-species similarity and intra-species variations. We constructed a dataset of 8 mosquito species that contains about 3,600 mosquito images of various poses and deformation conditions typically found in field-captured specimens. Despite this high inter-species similarity and various sample conditions, our results demonstrate that more than 97% accuracy can be achieved if several techniques, such as data augmentation and the fine-tuning of general features, are applied effectively to address data-scarcity problems. Further, we analyzed how this high classification accuracy can be achieved by localizing hot discriminative regions used by deep learning models. Our results show deep learning models learn similar discriminators from body areas of mosquitoes that are used by human experts for morphological diagnosis. We anticipate that our dataset, training methods, and results will inspire further research in the classification of vector mosquitoes.

More research is required to improve the accuracy of this automated identification work. First of all, we plan to expand the dataset to include more extensive and fine-grained set of mosquito species in various conditions (geographical distributions, life stages, blood-fed states, etc.) With such extensive and detailed dataset, we are particularly interested in classifying more similar and cryptic mosquito species. For example, in our current work, we grouped the *Anopheles* genus into a single class without further taxonomic separation since the species in *Anopheles* genus are extremely similar morphologically and can be further classified by careful examination on wing venation characters amongst *Anopheles* species complex. Although a few automated mosquito capture and monitoring systems are available at present for remote monitoring of mosquitoes in the field, these systems need accurate and rapid automatic identification in the first place. For further study, our current algorithm of identifying mosquitoes can be applied to developing in-field devices that monitor and classify mosquito species, which will shed light on real-time monitoring of mosquito species.

## Data Availability

The dataset and source codes for this work are publicly available through the first author’s GitHub repository: https://github.com/jypark1994/MosquitoDL.
